# Effects of different manufacturing techniques on the performance of planar antennas

**DOI:** 10.1038/s41598-023-49726-6

**Published:** 2023-12-15

**Authors:** Justina Žemgulytė, Modestas Sadauskas, Paulius Ragulis, Romualdas Trusovas, Karolis Ratautas, Rimantas Simniškis, Žilvinas Kancleris, Gediminas Račiukaitis

**Affiliations:** 1 Department of Physical Technologies, Center for Physical Sciences and Technology, Sauletekio Ave. 3, 10257 Vilnius, Lithuania; 2 Department of Laser Technologies, Center for Physical Sciences and Technology, Sauletekio Ave. 3, 10257 Vilnius, Lithuania

**Keywords:** Electrical and electronic engineering, Applied physics, Devices for energy harvesting

## Abstract

This study investigates antenna performance based on its manufacturing process. Two types of planar antennas are manufactured on FR-4 dielectric using three different techniques: traditional lithography, laser ablation, and the novel SSAIL (selective surface activation by laser) technique. Various characteristics, such as reflection coefficient, gain, half-power beamwidth, and surface conductivity, are measured to compare the results. These findings offer invaluable insights for choosing the most suitable antenna manufacturing technique, particularly since the SSAIL technique has not been previously compared to alternative methods in the context of antenna production. In both types of antennas, the highest gain is achieved using laser ablation, with the slot-loaded patch antenna reaching 8.5 dBi and the Yagi-Uda antenna reaching 9.76 dBi. Antennas manufactured using SSAIL technology are notable for their excellent resolution and usefulness in constructing structures on non-metallized dielectrics.

## Introduction

In the industry, microstrip structures on FR-4 or similar substrates are typically produced by acid etching, a tightly controlled and optimized process to ensure that every manufactured product is identical. In scientific work, the method of antenna fabrication is often determined by the tools available in the laboratory and is often not described in publications. The most common methods for fabricating microstrip structures are lithography^1,2^ and computer numerical control (CNC) milling^3^. With the emergence of a growing number of new techniques for fabricating microstrip structures, many scientific papers compare and deeply analyze various techniques^4–6^. For example, much attention is given to techniques for fabricating textile antennas^7–9^, metallization techniques for printed antennas^10–12^, additive manufacturing techniques^13^, and CNC milling^14–16^. However, lithography is still the most widespread manufacturing technique for traditional planar antennas. New technologies such as Selective Surface Activation Induced by Laser (SSAIL)^17^ and laser ablation are being developed in the FTMC Department of Laser Technologies. These technologies could be applied to antenna fabrication^18,19^ or even replace the techniques currently used in the industry. However, the parameters of antennas fabricated using these techniques have not been thoroughly investigated and there is an absence of such comparative analysis in scientific literature.

In this paper, for the first time, three manufacturing techniques were investigated and compared: traditional lithography, laser ablation, and the SSAIL technique. The most common method is lithography, mainly due to the low costs of the necessary chemicals. Laser ablation has limitations in terms of laser resolution, but it is less time-consuming as it is mostly automated and provides better resolution compared to CNC milling. Although the novel SSAIL technology has not been widely adopted, it offers appealing advantages over other techniques. With SSAIL, it becomes possible to selectively deposit metals on planar or even 3D structures made of various materials, ranging from standard dielectrics and ceramics to flexible polymers.

Research findings could also help to design a highly efficient and well-tuned antenna for energy harvesting. Radiofrequency energy harvesting is a fairly new and fast-growing field. To make a good energy harvester it is necessary to create system components with very low losses, which can be accomplished by producing system components on a single dielectric plate. By understanding the key parameters, materials, and processes that influence antenna performance, manufacturers and researchers can make informed decisions to enhance their antenna production’s quality, reliability, and cost-effectiveness, ultimately leading to improved wireless communication systems and technology.

The article is structured as follows. Section “Antennas” presents the antennas under investigation. The three manufacturing methods, lithography, laser ablation, and SSAIL, are described in Section “Manufacturing”. In Section “Antenna characterization” the parameters employed for the characterization of antennas are presented. Measurement results and their comparison are reported in Section “Results and comparison”. Finally, in the last section, the “Conclusions” are drawn. In the supplementary material, one can find some details of antenna characteristics.

## Antennas

**Figure 1:**
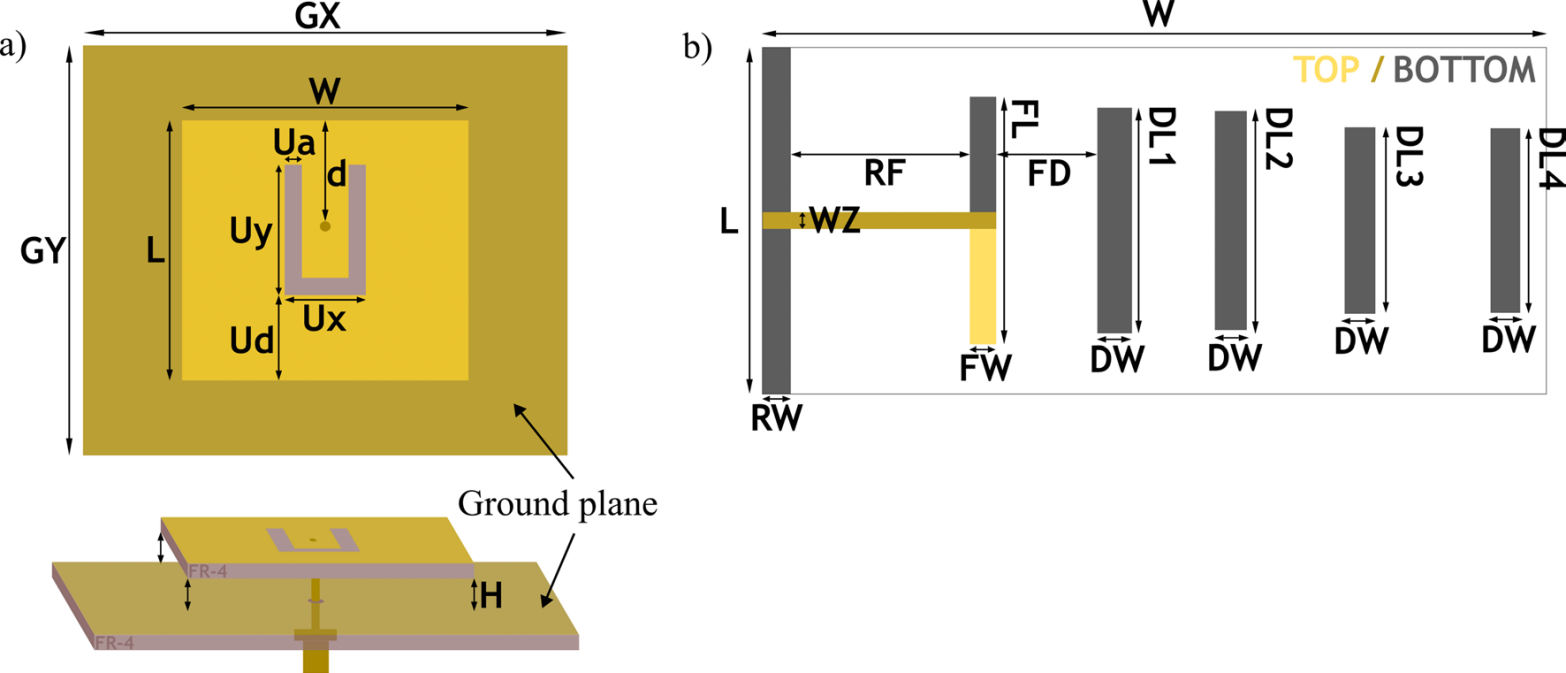
Antenna design, (**a**) - slot-loaded patch antenna, (**b**) - Yagi-Uda antenna.

In references^20,21^, a wide band slot-loaded patch antenna suitable for a low-power wireless transmission system is discussed. There are many configurations and types of slots that can be combined with the simple patch antenna to influence the antenna operation^22^. In our case, having a single operating frequency range that would cover the WiFi 2.4 GHz frequency band is desirable. We designed a similar U-shaped slot-loaded patch antenna (further called a slotted antenna) which is depicted in Fig. 1a. It comprises a radiating patch with a U-shaped slot, a dielectric (air), and a ground plane. For the radiating patch, FR-4 material with a dielectric constant of 4.3 and a thickness of 1.45 mm is utilized. One side of the FR-4 material is covered with a 0.05 mm thick copper layer. In the case of the SSAIL technology, the metal layer was grown, leaving a U-shaped vacant space. An additional 8 mm of air gap is included between the radiating plate and the ground layer to enhance gain and antenna efficiency. The resonance frequency of the slotted antenna can be adjusted to the desired frequency by modifying the patch length *L* and the air gap thickness *H*. The parameters of the slotted antenna are presented in Table 1.

**Table 1:** Slot-loaded patch antenna dimensions (mm).

W	L	H	G$_X$	G$_Y$	d	U$_a$	U$_d$	U$_x$	U$_y$
49.6	45	8	84	71	18.4	3	14.7	14.1	22.6

**Figure 2:**
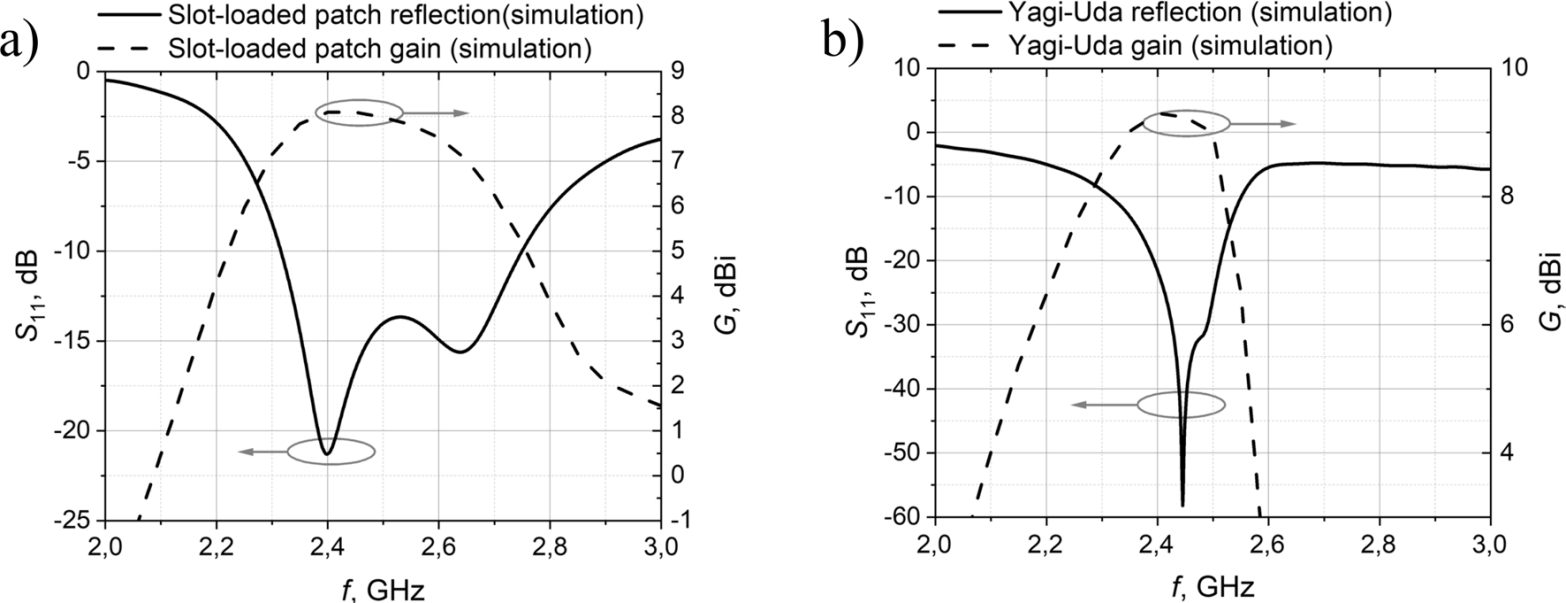
Simulated antenna reflection and gain characteristics, (**a**) - slot-loaded patch antenna, (**b**) - Yagi-Uda antenna.

In this study, the antenna was optimized to operate at the WiFi 2.4 GHz frequency. The reflection coefficient and gain parameters were computed using the finite difference time domain (FDTD) method. The operating frequency range spans from 2.31 to 2.75 GHz. The antenna’s half-power beam width is 73$^\circ$, achieving a maximum gain of 8.1 dBi at 2.45 GHz (see Fig. 2a).

**Table 2:** Yagi-Uda antenna dimensions (mm).

W	L	WZ	FL	RW	FW	DW	RF	FD	DL1	DL2	DL3	DL4
136	50	2.9	42.9	4.9	4.6	6	27.5	13.5	39.1	37.9	32.3	32

For energy harvesting purposes, high gain is sometimes more important than a wide operating frequency range, particularly when a dedicated power source like an internet router is used. An example of such a case is presented in^23^, where a wireless food assessment system is developed. This system uses a directive high-gain Yagi-Uda antenna for energy harvesting from a dedicated power source. Consequently, the second antenna chosen for this work is also a Yagi-Uda antenna. In this type of antenna, the most critical parameter influencing the resonant frequency is the dipole length (FL) (see Fig. 1b). Behind the dipole, a reflector is positioned to minimize back lobe radiation. Additionally, three additional directors are employed in the antenna design to achieve optimal gain. Finally, the antenna excitation is modified to achieve a phase delay between the two dipole arms without needing a balun. The operating frequency range of the Yagi-Uda antenna is from 2.3 to 2.55 GHz, with a maximum antenna gain of 9.3 dBi at 2.4 GHz and a half-power beam width of 59.9$^\circ$ (Fig. 2b). The geometric parameters of the Yagi-Uda antenna are presented in Table 2, and the antenna itself is depicted in Fig. 1b).

## Manufacturing

### Lithography

The template of the antenna was printed using a standard laser printer on a transparent sheet, which later served as a photomask for the photolithography process. The FR-4 panel was cut to size and prepared for lithography by treating the surface with an HCl solution to remove the native oxide layer and washing it with an alcohol solution. A positive photoresist layer (POSITIV20 by Kontakt Chemie®) was sprayed onto its surface by keeping the can at a 20–30 cm distance and making an “S” shaped motion for 0.2 s. The photoresist layer was left to dry in a heating oven at 70 $^\circ$C for about 20 minutes. The photoresist acted as a protective layer during subsequent steps. The photomask was attached to the plate, placed in a case with an ultraviolet lamp, and irradiated for 60 seconds. This exposure step helped transfer the design pattern onto the photoresist layer. For UV irradiation SMD Vishay®VLMU3100-GS08 LEDs with 405 nm wavelength and 120$^\circ$ viewing angle of half intensity were used. LEDs were positioned 40 mm away from the plate. Following the exposure, the plate was washed in a 10% Na$_2$SiO$_3$ solution. This solution removed the photoresist of the areas exposed to UV light. As the solution washed away the photoresist, the structure’s shape emerged on the plate surface. The washing process was continued until the photoresist in the exposed areas was completely removed, revealing the desired pattern. Etching was the next step, which used a 5% Na$_2$S$_2$O$_8$ solution. This etching solution selectively removed the copper layer, refining the structure and creating the final desired shape. The duration of the etching process could vary between 30 and 60 minutes, depending on the freshness of the solution used. Several factors influenced the achievable resolution of the manufactured structure. The ability to evenly coat the plate surface with the photoresist is essential for obtaining consistent results. The resolution of the photomask used during the exposure step determines the level of detail that could be transferred onto the photoresist. Additionally, the quality of the ultraviolet light source plays a role in achieving accurate and precise results. Paying attention to these factors helps ensure that the desired resolution is achieved during the manufacturing process.

### Direct ablation

Laser ablation is the process of removing material from the solid surface using laser irradiation. The depth of ablation depends on the optical properties of ablated material and various laser process parameters, like laser irradiation wavelength, pulse duration, pulse repetition rate, number of passes, and focusing conditions^24^, which define laser irradiation fluence on the material’s surface. Laser processes are widely used for various micromachining applications such as drilling^25^, cutting^26^, or synthesis of nanoparticles^27^. The laser ablation process has been demonstrated to be a versatile tool for antenna formation. Esakkimuthu et al. used a mode-locked laser for terahertz antenna applications^28^. A microsecond pulse UV laser for copper ablation on a flexible substrate for electronics applications was employed in^29^. Our approach includes a picosecond laser, as using shorter pulses enables the increase of ablation precision and a significant decrease in heat affected zone. The Nd:YVO4 picosecond laser Atlantic (Ekspla) with a pulse duration of 10 ps, a pulse repetition rate of 400 kHz–1 MHz, and a maximum average power of up to 60 W was used for the ablation technique. Specialized software was employed to process the design, allowing for customization of parameters such as scanning speed, the distance between lines, and optimal pathing to prevent thermal damage. In this particular case, the scanning speed was set to 1000 mm/s, and the distance between lines was set to 7 microns. During each scanning pass, approximately 1/6 of the metallization layer was removed. The scanning process was repeated up to 6 times to ensure complete removal.

### Antenna formation using SSAIL

**Figure 3:**
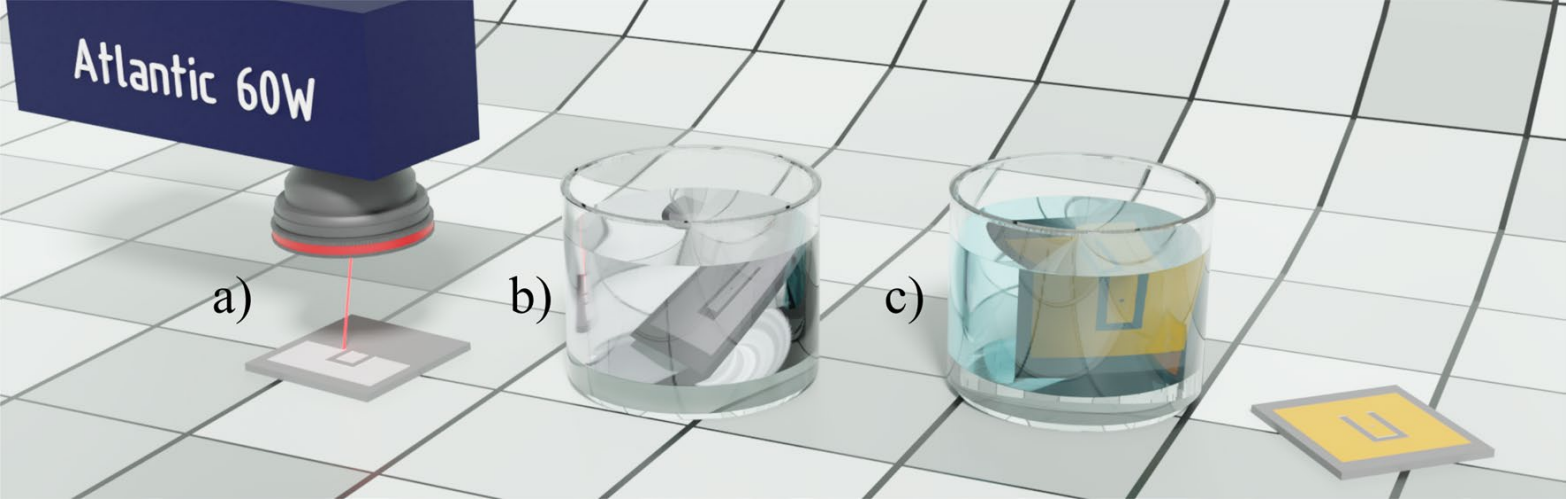
Steps of SSAIL process (**a**) - laser treatment, (**b**) - chemical activation, (**c**) - electroless copper deposition.

One of the most promising technologies for selective metal deposition is SSAIL^17,30,31^. The process involves laser modification of the polymer surface with a short-pulse laser, chemical activation of the laser-modified areas, and electroless metal deposition on the locally activated surface. The SSAIL technology consists of three chemical processing steps and one laser treatment. Ultrashort pulse laser-induced physical and chemical bonding between dielectric substrate material and plated metal guarantees strong adhesion. The process could be applied to almost any dielectric by adapting laser and chemical parameters. The nature of the process is not fully understood for every substrate, however, some of them, like polymers, have been more detailed investigated^17,30^, and it was shown that ultrashort laser pulses induced chemical changes in the irradiated surface by breaking chemical bonds and forming aldehyde compounds. These compounds participate in the catalyst formation process after chemical activation^30^. SSAIL technology offers high-speed laser modification for high-performance polymeric materials such as polyether ether ketone and liquid crystal polymer. In this work, we are applying the method on standard material FR 4 used for PCB manufacturing. For the SSAIL procedure, the same laser was used as in the case of direct ablation (see Section “Direct ablation”). A pulse picker was employed to adjust a lower frequency regime. The laser beam was translated with a galvanometric scanner (Scanlab AG). The F-theta lens of 160 mm focal length was used to focus the laser beam on the surface of the substrate. The laser beam was scanned over the area to be metallized by hatching–overlapping parallel lines (Fig. 3a). The focused laser beam spot size was 25 $\upmu$m in diameter (Gaussian intensity level $1/e^2$).

After the laser treatment, samples were washed with ethanol 99.8% (Sigma-Aldrich) and rinsed with distilled water afterwards. A highly diluted silver nitrate (Sigma-Aldrich) solution ( 10-5 M) was used for chemical activation (Fig. 3b). Finally, the electroless copper deposition was performed for 30 min at 30 $^\circ$C (Fig. 3c). The copper plating bath contained copper (II) sulfate pentahydrate (0.12 M), formaldehyde (0.3 M), sodium hydroxide (1.2 M), sodium carbonate (0.3 M), and sodium-potassium tartrate (0.35) (all Sigma-Aldrich) and pH = 12.7.

Firstly, different laser regimes were investigated by changing pulse repetition rate frequency from 10 kHz to 400 kHz, pulse energy from 5 to 50 $\mu$J, and laser writing speed from 50 to 2000 mm/s. By changing those parameters, the square-sized areas of 20x50 mm were scanned with a laser beam on the FR4 surface. After the specimen was activated and plated, including all the steps described above, each plated area was checked with an optical microscope for plating selectivity. The best laser regime was selected by examining the over-plating of copper outside of the laser-activated area. Sheet resistance was also measured using the four-probe method^32^.

## Antenna characterization

Several parameters were measured and analyzed to compare the manufactured slotted and Yagi-Uda antennas. These parameters include: reflection coefficient, operating frequency, maximum gain, half-power beam width, deviation of geometric parameters, and surface conductivity.

In addition to these performance parameters, other factors such as quality/defects, manufacturing time, and manufacturing costs have also been considered. Quality and defects refer to the overall reliability and robustness of the antenna while manufacturing time and costs determine the efficiency and affordability of the manufacturing process.

By evaluating and comparing these parameters and variables, one can make informed decisions about the performance, feasibility, and practicality of the slotted and Yagi-Uda antennas in different applications.

**Figure 4:**
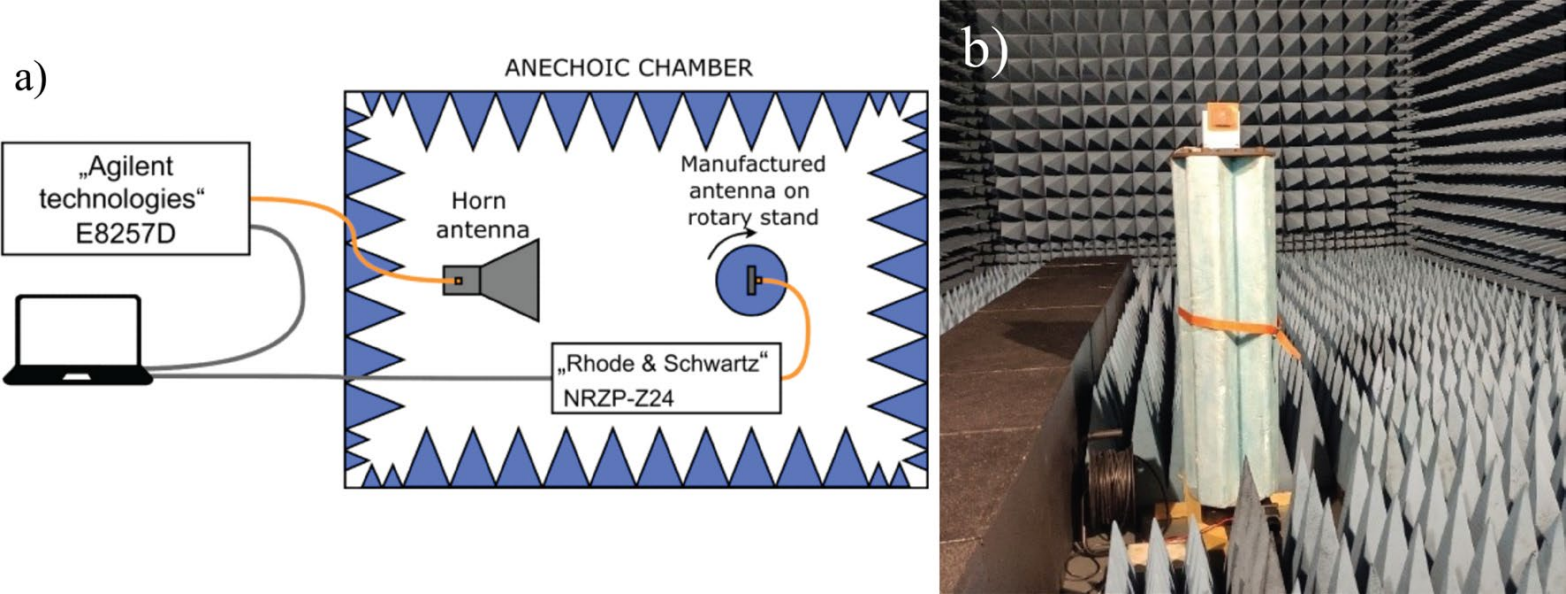
Measurement setup in an anechoic chamber (**a**) measurement scheme (**b**) rotary stand with slot-loaded patch antenna.

The gain and directivity characteristics of the antennas were measured in a microwave anechoic chamber using the following measurement setup, as explained in Fig. 4. The antenna under test was placed on a rotating stand, enabling it to be rotated 360$^\circ$. The rotation stage was controlled by a stepping motor. A calibrated Rohde & Schwarz NRZP-Z24 power meter was connected to the antenna’s excitation port. The power meter could measure power from -42 dBm to 45 dBm across frequencies ranging from 10 MHz to 18 GHz. It is connected to a computer via a USB cable. A broadband horn antenna with known specifications was positioned 2 meters from the antenna under test. A coaxial cable connected the horn antenna to an Agilent Technologies E8257D analog signal generator. A measurement program controlled the rotation stand, power meter, and signal generator. To measure the frequency response of the antenna, the power of the signal fed to the horn antenna was kept constant at 20 dBm, while the frequency was varied from 2 GHz to 3 GHz. This allowed for the measurement of gain at different frequencies within that range. To measure the gain dependence on the angle, the power was set to 20 dBm, the frequency was locked at 2.45 GHz, and the antenna under test was rotated from 0$^\circ$ to 360$^\circ$ in 1$^\circ$ increments. The gain was then calculated using Eq. (1):

1 $$\begin{aligned} G = \frac{P\cdot \left( 4 \cdot \pi \cdot R \right) ^2 }{P_{tr} \cdot G_{tr} \cdot \lambda ^2} . \end{aligned}$$

here *P* - is the measured power, *R* - is the distance between antennas, *P*$_{tr}$ - is the transmitted power, *G*$_{tr}$ - is the transmitting antenna gain, $\lambda$ - is a wavelength.

The deviation of antenna geometric parameters was measured using an electronic caliper. This instrument allows for precise measurements of dimensions such as length, width, and height, ensuring that the manufactured antennas closely match the intended design specifications. To measure the metallization thickness, a profilometer was used. The profilometer was specifically used to determine the thickness of the metallization layer on the antenna surface after the SSAIL or laser ablation procedure. Surface conductivity was measured using the 4-probe technique, which involves taking the average of five measurements in different areas of the antenna surface. This technique provides an accurate assessment of the surface conductivity. A comprehensive evaluation of the antennas’ performance and manufacturing quality could be achieved by employing these measurement techniques and obtaining information about structural accuracy, metallization thickness, and surface conductivity.

## Results and comparison

**Table 3:** Measured parameter deviation from the initial design (mm).

Slot-loaded patch antenna	Lithography	Ablation	SSAIL
Patch length (*L)*	$-$1.59 - $-$0.4	$-$0.5 - 0.2	$-$0.1 - $-$0.01
Patch width (*W)*	$-$1.71 - $-$0.2	$-$0.5 - 0.1	$-$0.1 - 0
Slot width (*Ua1)*	$-$0.17 - 0.23	$-$0.18 - 0.19	$-$0.1 - 0.1
Excitation (*d)*	**-**0.68 - $-$0.56	0 - 0.1	0.2 - 0.6
Yagi-Uda antenna			
Reflector length (*L)*	$-$0.67 - 0.56	$-$0.7 - $-$0.1	$-$0.18 - 0.1
Excitation line width (*WZ)*	$-$0.35 - 0.03	$-$0.25 - $-$0.06	$-$0.3 - 0
Dipole length (*FL)*	$-$0.25 - 0.1	$-$0.3 - 0	$-$0.5 - 0.2
Dipole width (*FW)*	$-$0.35 - 0.15	$-$0.17 - $-$0.1	$-$0.18 - 0

**Figure 5:**
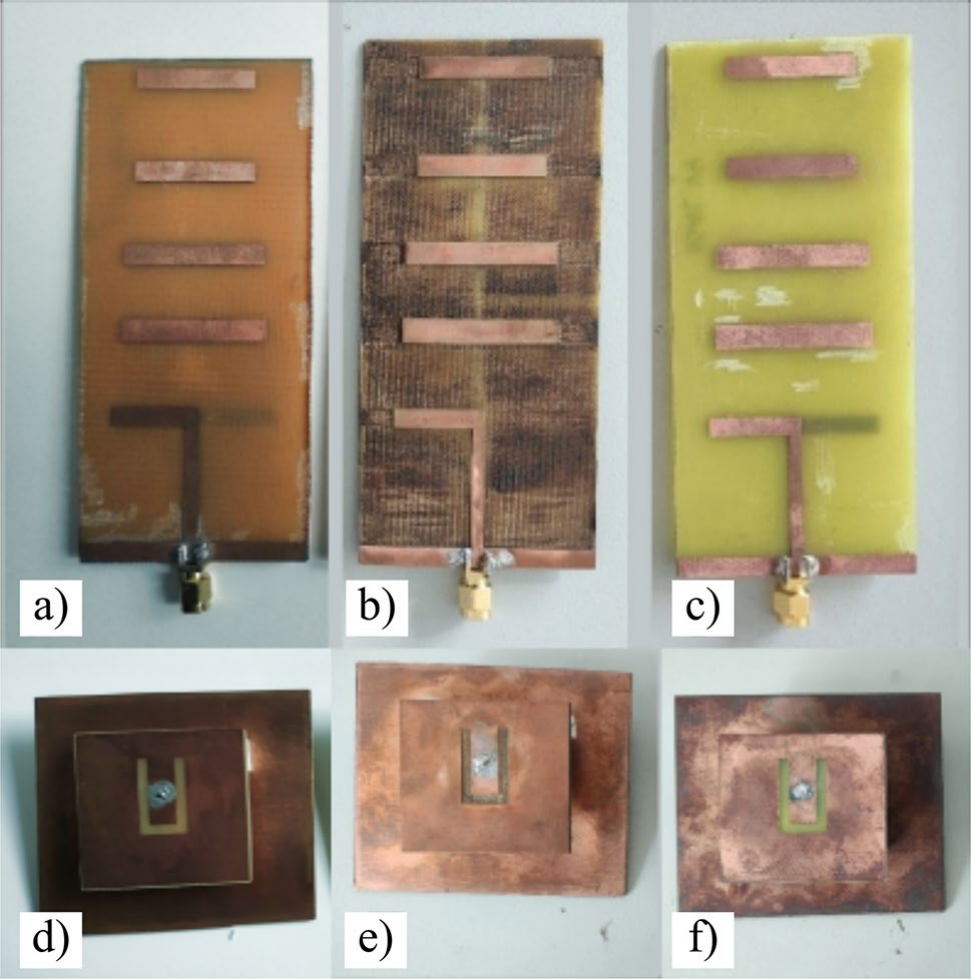
Pictures of slot-loaded patch and Yagi-Uda antennas manufactured using three techniques (**a**,**d**) - lithography, (**b**,**e**) - laser ablation, (**c**,**f**) - SSAIL).

After manufacturing three antennas for each manufacturing method (lithography, ablation, SSAIL) (Fig. 5) using the described methods, structure dimensions are measured and compared (see Table 3). The measured parameters, such as patch length, patch width, excitation, and distance to the ground plane, are mainly subject to human error, resulting in noticeable variations. On the other hand, parameters such as slot width, reflector width and length, excitation line width, dipole length, and width exhibit significantly less deviation. The deviations observed in these parameters can be attributed to factors such as under-etching or over-etching and poor mask quality. Notably, when comparing the manufacturing methods, the use of ablation and SSAIL techniques resulted in smaller structure deviations compared to lithography, except for the Yagi-Uda dipole length in the case of SSAIL.

**Figure 6:**
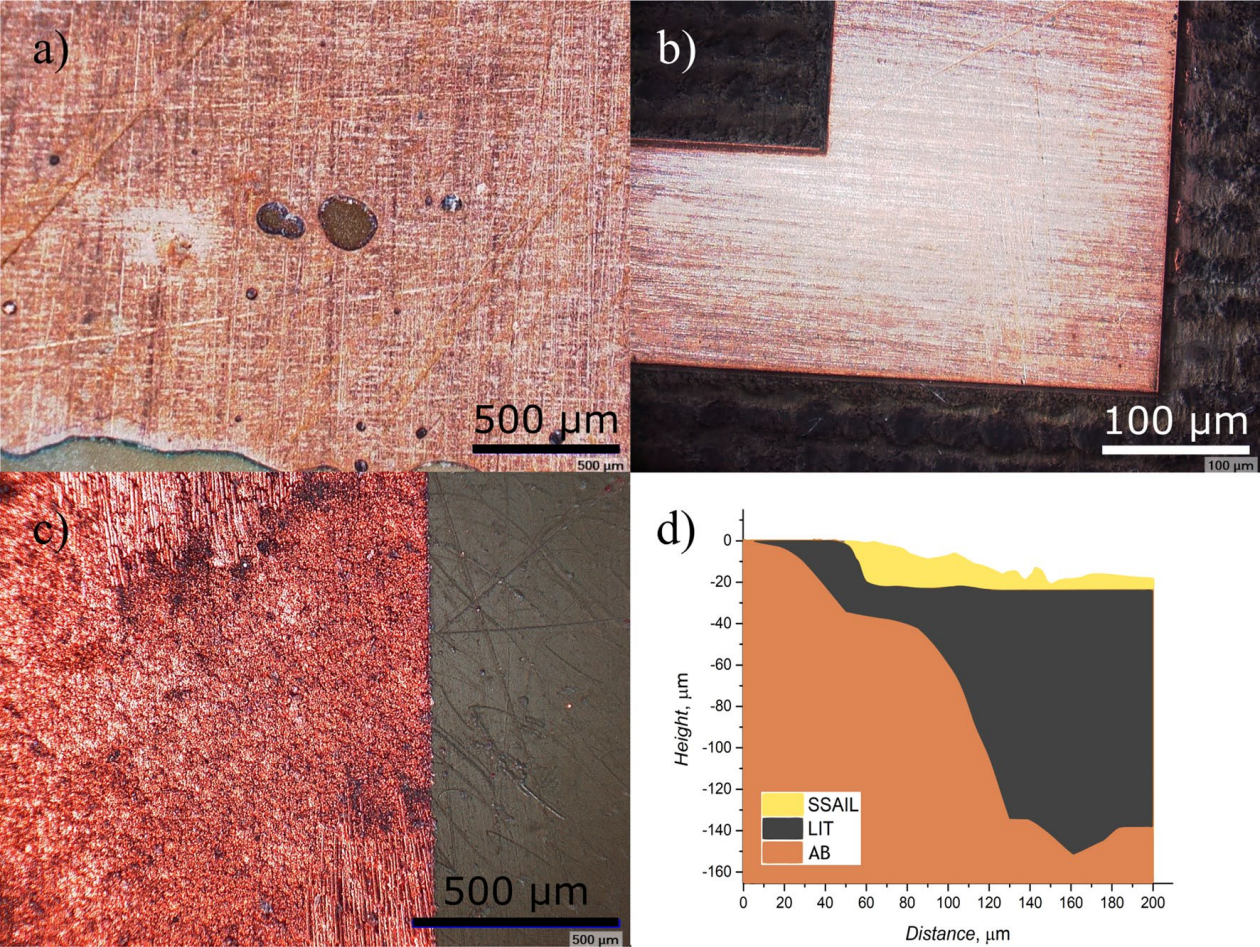
Observed defects in manufactured antennas, (**a**) - Lithography, (**b**) - laser ablation, (**c**) - SSAIL, (**d**) - surface measurement using a profilometer.

During the examination of the antenna surface under a microscope, shown in Fig. 6, several other defects were discovered. For example, antennas manufactured using the lithography method (Fig. 6a) exhibited small holes in the metallization layer, and the metallic edges appeared uneven. On the other hand, antennas manufactured using laser ablation technology had the cleanest edges, but a common issue observed was the burns of the dielectric plate during the process (Fig. 6b). This resulted in forming a graphitic layer^33^, which exhibited surface conductivity ranging from 10 $\Omega$ to 10 k$\Omega$. The presence of this carbon layer significantly impaired the antenna’s ability to radiate, requiring it to be scraped off to restore proper antenna operation. However, this scraping process also leads to changes in substrate thickness, which, as mentioned in^34^, can impact antenna parameters. While examining the antennas manufactured using SSAIL, poorly metallized zones were detected on some Yagi-Uda antennas. Additionally, the Yagi-Uda antenna surface was frequently contaminated with small metal fragments (Fig. 6c), which could potentially indicate an error in the surface activation step of the SSAIL process. These defects and irregularities observed during the microscopic examination provide important insights into the manufacturing process and highlight areas that require further optimization to ensure consistent and reliable antenna performance.

Upon closer examination of the dielectric surface height (Fig. 6d), in the sample made using laser ablation, two distinct steps were clearly visible. The first step was located right at the edge of the metallization, indicating the boundary between the metal and the dielectric material. The second step was observed where the laser had cut away into the dielectric material, creating a noticeable change in surface height. In contrast, the SSAIL sample did not exhibit an obvious step in the dielectric surface height. This is because the SSAIL process involves treating the surface with a laser before plating. This treatment creates a hollow hole where the metal is subsequently grown. As a result, there was no distinct step at the edge of the metallization in the SSAIL sample. These observations highlighted the differences in the manufacturing processes between laser ablation and SSAIL. The presence of steps in the laser ablation sample and the absence of such steps in the SSAIL sample have implications for the overall surface topology and can potentially affect the performance and characteristics of the antennas. Using simulation software, the dependence of antenna operation on certain defects was examined further, the findings are shown in Section 2 of the supplementary material.

**Figure 7:**
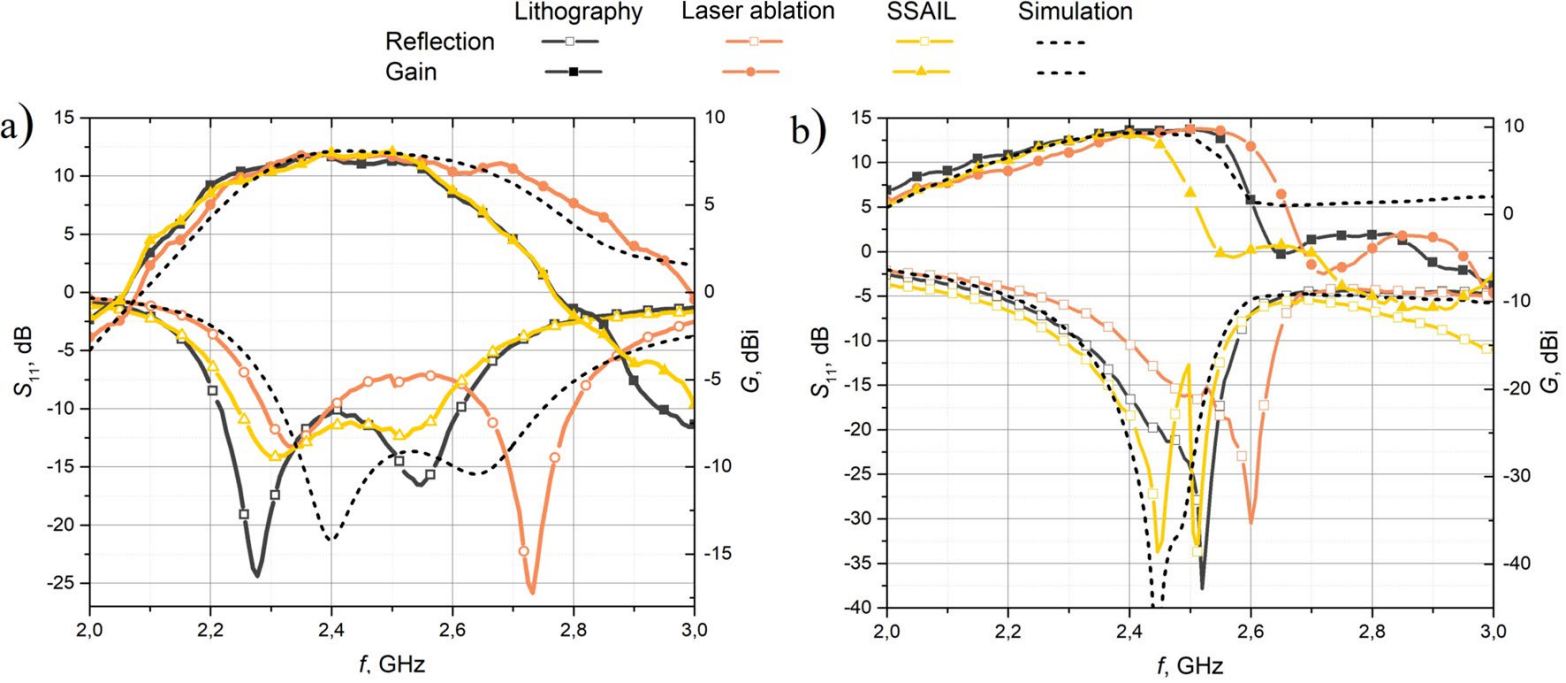
Best antenna results out of all manufacturing methods, reflection coefficient and gain: (**a**) - slot-loaded patch antenna, (**b**) - Yagi-Uda antenna.

Fig. 7 provides an overview of the gain and reflection characteristics of the best antennas manufactured using the three different methods. Several observations could be made upon analyzing the slotted antenna’s reflection plot. Firstly, the ablated antenna exhibits a center frequency that aligns well with the simulation. However, the reflection between 2.4 GHz and 2.65 GHz exceeds the $-$10 dB limit, indicating poor impedance matching. For the antennas manufactured using lithography and SSAIL, the operating bands are narrower, particularly on the higher frequency side, and they are shifted towards lower frequencies. This trend is also evident in the frequency response of these antennas. Regarding gain, only a slight variation is observed between the different manufacturing methods. The lithography antenna has a gain of 7.9 dBi, the ablation antenna shows a gain of 8.5 dBi, and the SSAIL antenna demonstrates a gain of 8 dBi. These findings suggest that while the gain remains relatively consistent across the different methods, there are differences in the impedance matching and frequency response.

**Table 4:** Measurements of all manufactured antennas.

Antenna Nr.	Cost, Eur	Time	Quality/defects	Operating frequency (S$_{11}<$-10 dB), GHz	Maximum gain, dBi	Half power beam width (3 dB),$^\circ$	Surface conductivity, MS/m
SLOT-LOADED PATCH ANTENNA							
0	–	–	–	2.317–2.754	8.1	73	58
1	0.5	1h 40 min	Coarse metallization edge, holes (500 $\upmu$m)	2.22–2.61	7.9	66	9.456
2				2.25–2.65	7.2	65	13.789
3				2.245–2.65	7.38	65	18.912
1	5 + laser	15 min	Dielectric height reduction, burns on FR-4 surface	2.29–2.39. 2.66–2.79	7.95	67	13.508
2				2.31–2.46. 2.64–2.81	8.5	68	12.035
1	6 +laser	1 h 30 min	Uneven metallization layer, under-plated zones	2.245-2.58	8	68	17.651
2				2.14–2.285	8	68	1.324
YAGI-UDA ANTENNA							
0	–	–	–	2.32–2.55	9.3	60	58
1	1.7	3 h 30 min	Coarse metallization edge, holes (500 $\upmu$m)	2.36-2.58	9.6	61	16.548
2				2.3–2.58	9.3	67	8.274
3				2.32–2.58	9.7	66	7.355
1	5 +laser	5 h 20 min	Dielectric height reduction, burns on FR-4 surface	2.44-2.72	9.33	65	11.032
2				2.48–2.75	8.69	70	7.355
3				2.4–2.64	9.76	61	4.903
1	6 +laser	2 h	Uneven metallization layer, under-plated zones, dielectric surface contamination	2.34-2.55	7.88	42	3.309
2				2.27–2.52	5.88	54	4.903
3				2.29–2.56	9.07	34	18.912
4				2.37-2.69	8.52	42	33.095

Analyzing the reflection plot of the Yagi-Uda antenna (Fig. 7b), some noteworthy observations could be made. The SSAIL antenna best matches the simulated operation frequency, indicating accurate fabrication. The lithography antenna exhibits a slight shift towards higher frequencies, while the ablation antenna shows a more significant shift in the same direction. Regarding frequency response, the lithography antenna aligns closely with the simulation, suggesting good overall performance. The frequency response of the ablation antenna is slightly shifted towards higher frequencies, while the SSAIL antenna shows a narrower gain band, particularly on the higher frequency side. Finally, the maximum gain achieved by the antennas only varies slightly between the different manufacturing methods. The lithography antenna achieves a maximum gain of 9.7 dBi, the ablation antenna reaches 9.76 dBi, and the SSAIL antenna attains 9.07 dBi. These findings indicate that while the lithography antenna shows better frequency response alignment with the simulation, the SSAIL antenna demonstrates a closer match to the desired operating frequency. Additionally, measurement results of antenna radiation patterns can be found in the Supplementary material Section 1.

Table 4 provides a comprehensive overview of the results obtained from all manufactured antennas, allowing for comparative analysis. Several notable findings can be observed. Antennas manufactured using lithography exhibit the lowest deviation in operation frequency. This indicates better accuracy in achieving the desired frequency compared to the other methods. The lithography method is found to be more time-consuming when fabricating double-sided structures. This suggests that the process of lithography for such structures requires additional time and effort. Antennas manufactured with laser ablation showed a slight shift in operation frequency, specifically towards the higher frequency side. This phenomenon could be attributed to increased dielectric conductivity and decreased dielectric thickness, affecting the antenna’s performance. Laser ablation proved to be impractical for structures that require extensive removal of metallization. This limitation indicates that laser ablation may not be suitable for antennas with complex metallization patterns or those that involve substantial material removal. Antennas manufactured using the SSAIL method demonstrated favorable results for slotted antennas, with good alignment to the desired operating frequency. However, the Yagi-Uda antenna manufactured using SSAIL showed a smaller gain compared to the other methods. This discrepancy in gain could be attributed to uneven metallization, which could be related to the larger surface area, indicating the need for further refinement in the SSAIL process for Yagi-Uda type antennas. To investigate the potential impact of metallization quality on antenna characteristics, an additional experiment was conducted. Yagi-Uda antennas underwent tin-plating with a thin layer of solder (refer to results in the supplementary material, section 3). Insignificant deviations in reflection parameters were observed in the cases of lithography and laser ablation. However, in the case of SSAIL, a small frequency shift to higher frequencies was noted when conductivity was increased. Therefore, we can deduce that insufficient conductivity of SSAIL metallization could be the reason for the mismatch with the simulation results. It is important to note that in SSAIL, the laser surface processing lasted only 1-3 minutes, while the chemical treatment took the longest. This means that production quantity over time can be greatly increased because several samples can be chemically treated at the same time. The cost was calculated, considering the antenna surface area, but without factoring in the expenses associated with the required equipment.

## Conclusions

In conclusion, each of the manufacturing methods studied has advantages and limitations for antenna fabrication. Despite being cost-effective, photolithography may exhibit issues with hole formation in the metallization layer, which can impact antenna performance at higher frequencies. Therefore, it is best suited for small and medium-sized structures (5–100 mm$^2$). Laser ablation, on the other hand, offers precise edge quality but requires an experienced operator to avoid damaging the dielectric surface, because reducing dielectric height and changing surface conductivity of the dielectric surface can have an impact on antenna operation frequency and its radiation characteristics. Therefore, it is most suitable for structures that require only a small section of metallization to be removed. Finally, SSAIL stands out for its ability to achieve high resolution and is effective for fabricating structures on non-metallized dielectrics, especially when the metallization area is small. A few additional things need to be considered when using this method for antenna fabrication. Firstly, the metallization layer is buried into the dielectric, therefore antenna model has to be changed accordingly. Secondly, special care is required to achieve a uniform metal layer and high metal surface conductivity, because these parameters have an impact on antenna operation. However, it is worth noting that the SSAIL technology is still relatively new and may not be widely accessible due to its requirement for expensive equipment. Factors such as cost, resolution requirements, metallization area, and operator expertise should be carefully considered to determine the most appropriate manufacturing method for a specific antenna design. Additionally, ongoing research and development efforts in methods based on laser technologies hold the potential for further advancements and improvements in antenna manufacturing techniques.

### Supplementary Information

Supplementary Information.

## Data Availability

The datasets used and/or analysed during the current study are available from the corresponding author on reasonable request.
