# Supplementary material for: Effects of different manufacturing techniques on the performance of planar antennas
Source: Sci Rep. 2023 Dec 15;13:22510. doi: 10.1038/s41598-023-49726-6 (PMC10728079; doi:10.1038/s41598-023-49726-6)
Supplement: Supplementary file 1 — Supplementary Information. [file 41598_2023_49726_MOESM1_ESM.pdf]

Supplementary material

Antenna characteristics

In this supplementary material, we present some details of the manufactured antennas characteristics. The supplementary material is divided into three sections. The first section presents a comprehensive assessment of the directivity of antennas employing different manufacturing technologies. In the second chapter, we present the calculation results of the reflection coefficient accounting for different effects of manufacturing technology on metallization and dielectric properties. Finally, in the third section, we look into examination of the effect of tin plating on antenna parameters.

1. Antenna directivity

In this section, we present the results of antenna directivity measurements, comparing the radiation characteristics of Yagi-Uda and slot-loaded patch antennas manufactured using three distinct methods: traditional lithography, laser ablation, and the innovative SSAIL technique.

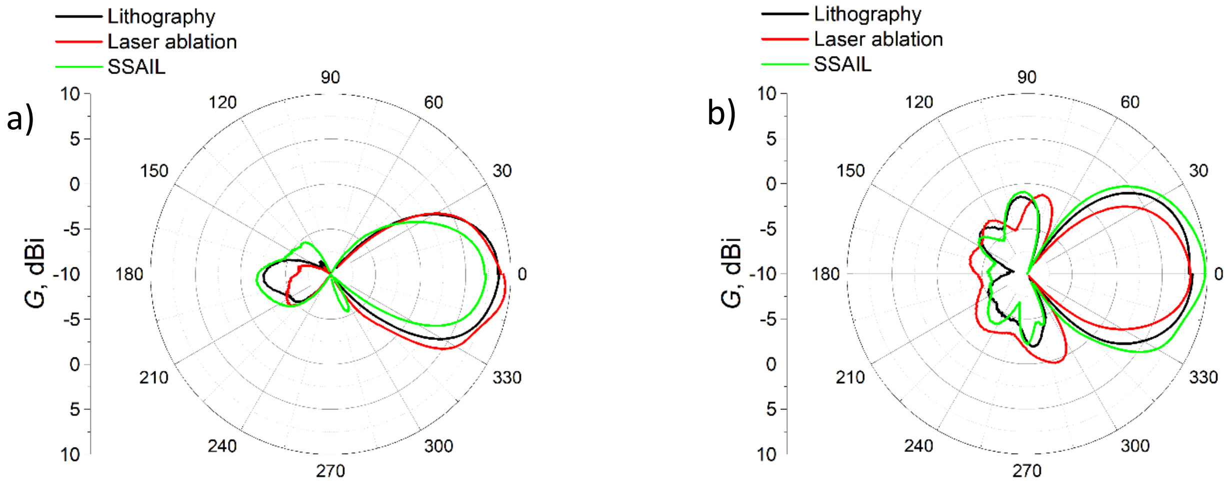

Fig. S1: Yagi-Uda antenna radiation characteristics at 2.45 GHz, a) - radiation pattern in E-plane, b) - radiation pattern in H-plane

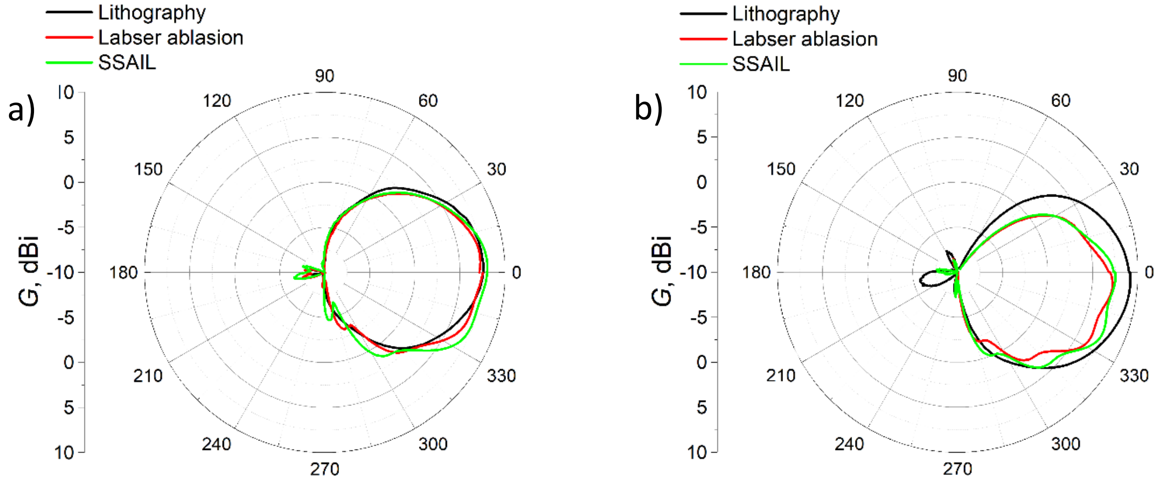

Fig. S2: Slot loaded patch antenna radiation characteristics at 2.45 GHz, a) - radiation pattern in E-plane, b) - radiation pattern in H-plane

Fig. S1 presents radiation characteristics of the Yagi-Uda antennas at 2.45 GHz. It can be seen that the directivity patterns across the manufacturing methods exhibit similarities, with minor variations that do not significantly impact the overall antenna's performance. The Half Power Beam Width (HPBW) values derived from these patterns are further detailed in Table 4 of the main article.

In Fig. S2, radiation characteristics of the slot-loaded patch antennas at 2.45 GHz are shown. The comparable trends in directivity among manufacturing methods persist, in slot-loaded patch antenna measurements.

2. Manufacturing influence on antenna characteristics

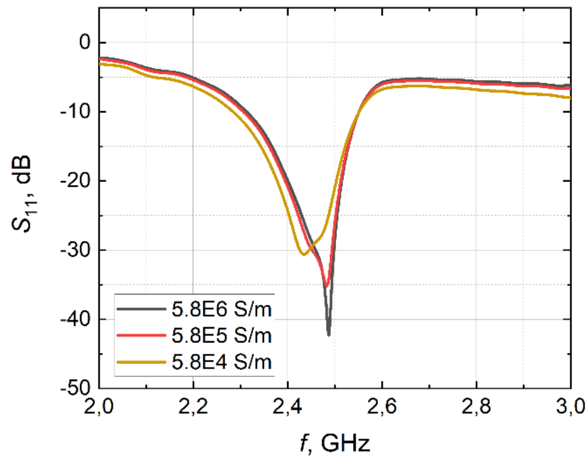

Fig. S3: Calculated dependence of reflection coefficient on frequency for Yagi-Uda antenna when changing specific conductivity of metallization (SSAIL case).

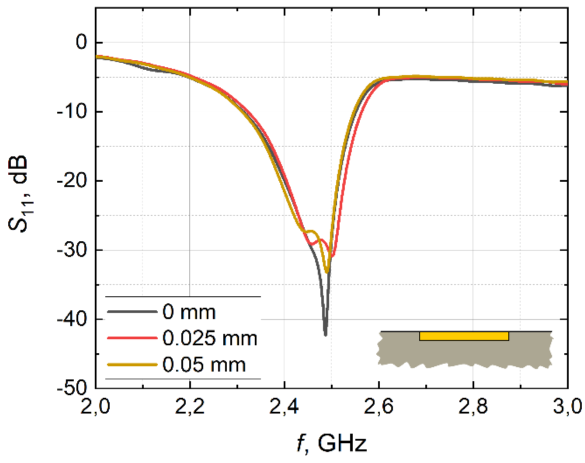

Fig. S4: Calculated dependence of reflection coefficient on frequency for Yagi-Uda antenna when changing metallization layer penetration into dielectric as a consequence of surface modification with a laser pulse (SSAIL case).

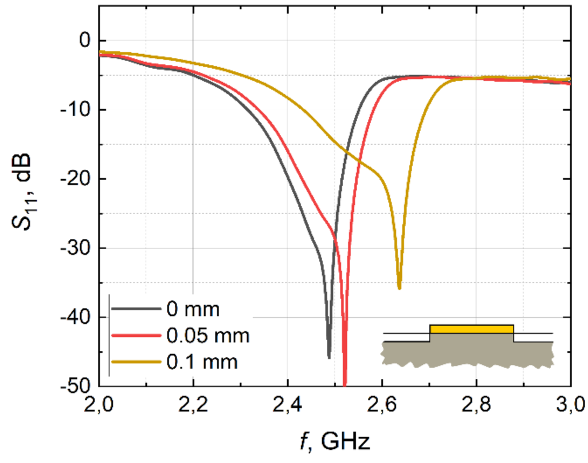

Fig. S5: Calculated dependence of reflection coefficient on frequency for Yagi-Uda antenna when dielectric height reduction is accounted for (laser ablation case).

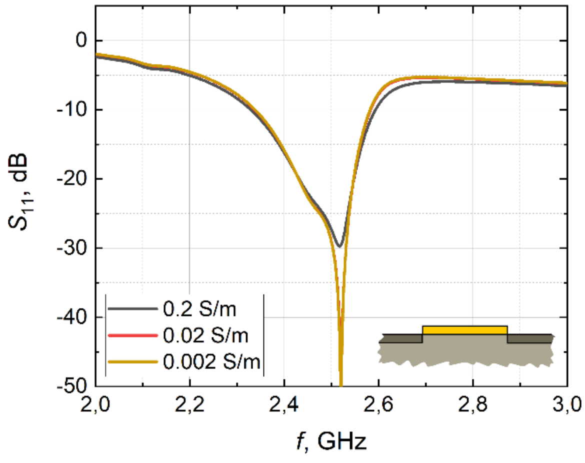

Fig. S6: Calculated dependence of reflection coefficient on frequency for Yagi-Uda antenna when different specific conductivity layer of 0.05 mm thickness is formed on the surface of dielectric material, simulating burned dielectric surface (laser ablation case).

In this section, we analyze the impact of manufacturing methods, specifically laser ablation and SSAIL, on antenna characteristics. Let's first consider potential manufacturing errors in each process. In SSAIL, two scenarios are likely: the conductivity of the SSAIL-produced metal layer could be lower than the bulk metal, and the metal layer might grow partially within the dielectric layer. For laser ablation, two plausible scenarios are likely to happen as well: the ablation process may remove not only the metallic layer but also part of the dielectric layer, and a partly conductive carbon coating could form during the process, influencing antenna performance.

Fig. S3 and Fig. S4 illustrate the influence of SSAIL manufacturing on the antenna reflection coefficient. Fig. S3 demonstrates the impact of metallization conductivity, revealing that decreasing conductivity affects only the depth of S_{11} parameters while leaving the central frequency unchanged. Fig. S4 explores the penetration of the metal layer into the dielectric, showing a similar effect. Comparing these results to the measurements presented in the main article (Fig. 7b), it becomes evident that these defects could be plausible explanations for the mismatch between simulations and measurements.

Fig. S5 and Fig. S6 examine the influence of laser ablation manufacturing on antenna reflection coefficient. Fig. S5 presents the effect of dielectric ablation along with the metallic layer, indicating that the removal of the dielectric layer shifts the center resonance to higher frequencies. In Fig. S6, the impact of conductive carbon formation in the ablation area is analyzed, showing that the center frequency remains constant while the depth of S_{11} at the resonance significantly reduces with an increase in the conductivity of the carbon layer. Once again, comparing these results to the measurements of the main article (Fig. 7b) suggests that these manufacturing defects could reasonably explain the shift in resonance frequencies and reduced reflectance observed in the manufactured antennas.

3. Yagi-Uda antenna tin-plating

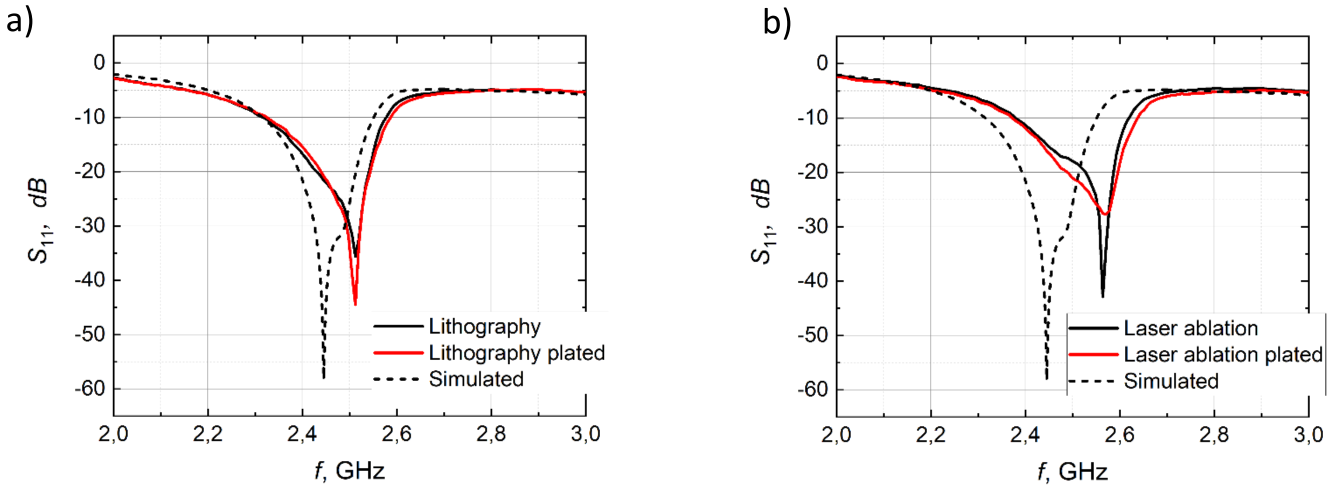

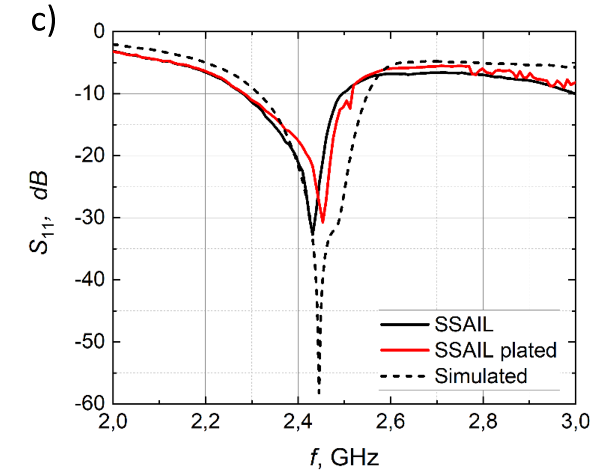

Fig. S7: Comparison of Yagi-Uda antenna reflection coefficients measured before and after tin-plating, using different manufacturing methods: a) lithography; b) laser ablation; c) SSAIL.

In this section, we analyze the influence of tin plating on Yagi-Uda antenna characteristics. Tin plating is occasionally employed when uncertainties arise regarding the quality of metallization. Upon examining the antennas through measurements and microscopic examination (see Fig. 6 in the main article), certain surface irregularities prompted us to explore the efficacy of tin plating on our manufactured antennas.

Fig. S7 provides a comparative analysis of the Yagi-Uda antenna's reflection coefficient before and after tin plating, considering different manufacturing methods. The outcomes reveal that, in the cases of lithography and laser ablation (Fig. S7 a) and Fig. S7 b)), there are no meaningful changes in the reflection coefficient. However, in the case

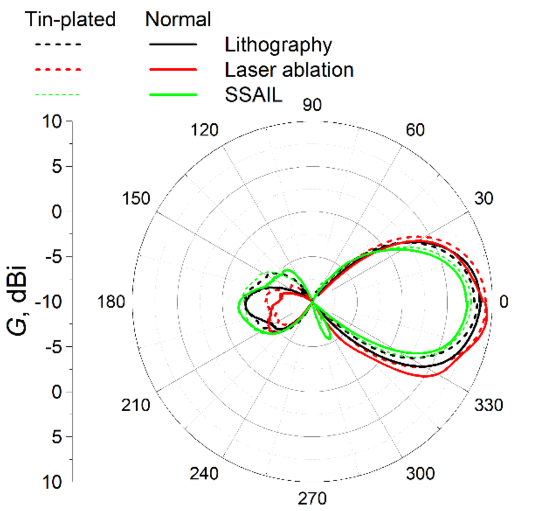

Fig. S8: Comparison of Yagi-Uda antenna radiation patterns measured in E-plane before and after tin-plating, using different manufacturing methods.

of SSAIL (Fig S7 c)), a slight frequency shift towards higher frequencies is evident after tin plating. As previously discussed, this shift may be linked to variations in metallization conductivity (Fig. S3).

Fig. S8 illustrates the comparison of the Yagi-Uda antenna's radiation pattern before and after tin plating. The results indicate that there is no significant difference in the radiation pattern between the pre- and post-tin plating conditions. This observation suggests that tin plating does not substantially alter the antenna's radiation characteristics.
